# The etiologic spectrum of head and neck squamous cell carcinoma in young patients

**DOI:** 10.18632/oncotarget.11265

**Published:** 2016-08-12

**Authors:** Xin Liu, Xiao-lei Gao, Xin-hua Liang, Ya-ling Tang

**Affiliations:** ^1^ State Key Laboratory of Oral Diseases West China Hospital of Stomatology, Sichuan University, Chengdu, Sichuan, People's Republic of China; ^2^ Department of Oral and Maxillofacial Surgery, West China Hospital of Stomatology, Sichuan University, Chengdu, Sichuan, People's Republic of China; ^3^ Department of Oral Pathology, West China Hospital of Stomatology, Sichuan University, Chengdu, Sichuan, People's Republic of China

**Keywords:** head and neck squamous cell carcinoma, young patient, chronic inflammation, HPV, immunity

## Abstract

Head and neck squamous cell carcinoma (HNSCC), accounting for more than 80% in head and neck malignancies, kills thousands of people a year in the world. Despite most of the patients are more than 45, and the occurrences of head and neck cancer shows a decreasing trend; however, horribly, the incidences of the patients under 45 years old is steadily increasing. Hence, it's of vital importance to get more pathogen information about risk factors of HNSCC in children and young adults. This review outlines the etiologic spectrum of HNSCC, especially oral/oropharyngeal squamous cell carcinoma, in patients under 45 years of age.

Head and neck squamous cell carcinoma (HNSCC), a group of related neoplasms that arise in the oral cavity, oropharynx, hypopharynx, and larynx, is the 6th most common cancer worldwide. Almost 650 000 new cases of head and neck cancer [1] and 300 000 deaths occur worldwide each year [2]. About 50-70% of head and neck cancers happened in males from 60 to 70, who have the habits of smoking and drinking [3–6], and the number of HNSCC is decreasing worldwide, however, epidemiological studies over last 20 years revealed that the overall incidence of young patients under 45 who encounter HNSCC, especially oral/oropharyngeal squamous cell carcinomas(OSCC/OPSCC), is growing steadily [4, 7–9]. Some reports suggest that about 6.7% of HNSCC patients are under 45 years of age, and 0.4-3.6% of patients are younger than 40 [10, 11]. The rising incidence is most strongly seen in developing countries like South and Southeast Asia and India, which is possibly due to their high production of areca nut [12–14]. In the Western world however, the incidence of young-onset HNSCC varies significantly between countries. several recent reports suggest that including Europe and the United States, the frequency of HNSCC has been decreasing over the past 30 years, while a nearly uniform rise is noticed in oral cavity SCC(OCSCC) and OPSCC, and the trends of young-onset HNSCC is disproportional to HNSCC patients above 45 years old [15–17]. Conway et al. noted carcinoma of the oral cavity and oropharyngeal region increasing in young white patients from 1990 to 1999 in the United Kingdom [16]. A study from the United States reported an increase in tongue carcinoma in patients from 18 to 44 in 1975-2007 [9](Table 1). Conversely, squamous cell carcinomas in laryngeal and hypopharyngeal regions in young patients are rare, account for approximately 10% and 1% of all young-onset HNSCC and the incidence remains quite stable these years [18, 19].

**Table 1:** Worldwide trends for HNSCC incidence in different age groups and sexes

Country	Author	Sitea	Years	Age	Sex	Trend	Additional
China	Chen et al. [14]	OC, OP, L	1981–2002	0–44	M + F	Increase	
USA	Schantz and Yu [17]	OT, Ph, L	1973–1997	<40	M + F	Increase	Significant(T)
USA	Shiboski et al. [7]	Tongue/Tonsil	1973–2001	20–44	M + F	Increase	
United Kingdom	Conway et al. [16]	OC, OP	1990–1999	<45	M + F	Increase	
USA	Patel et al. [9]	OT OC	1975–2007	18–44	F M	Increase Decrease	
USA	Muller et al. [20]	OC, OT	1971–2006	<40	M + F	Increase	
Netherlands	Braakhuis et al. [21]	OP	1989–2006	<45	M + F	Decrease	
Scandinavia	Annertz et al. [8]	T	1960–1994	20–39	M + F	Increase	M:5-fold F:6-fold
USA	Davis and Severson [22]	T	1973-1987	30-39	M	Increase	3-fold
Scotland	Macfarlane et al. [23]	OP	1970-1989	Young	M	Increase	

OC = oral cavity, OT = Oral Tongue, OP = Oropharynx, Ph = Pharynx, L = Larynx.

Cancer in the oral cavity generally predominates in younger patients, with the tongue being the most common subsite [24–27]. Compared with older patients, tongue cancer accounts for a larger proportion, while cancer in the floor of mouth accounts for a smaller proportion in younger patients [24]. In addition, Oropharyngeal cancer also accounts for a higher percentage of HNSCC in young patients compared with older patients [28].

Recent reports have demonstrated that the grade and combined stage are similar between young and old patients with SCC in oral cavity and tongue [24, 28, 29]. However, a number of studies have found that younger patients have a higher incidence of clinically detectable nodal metastases [18, 27, 28].

Moreover, studies show that there are no specific pathological characteristics of head and neck carcinomas in young adults. Sasaki et al. reported that the difference in lymph nodes between young and old population was not significant [3]. Mafi et al. found that there is little evidence that pathological features including tumor grade, lymph node status, tumor size, lymphovascular invasion, depth of tumor in younger individuals are different from older individuals [30].

Within the young subgroup, the rate of male and female patients is different. In the older group, approximately 70% of cases are males, while in the younger group shows men account for 50-65% of cases and the majority of young HNSCC patients without alcohol and tobacco consumption are females [31]. (Table 2 shows the clinical and histopathological characteristics of HNSCC of young and old patients)

**Table 2:** Summary of studies and cases reporting clinical and histopathological characteristics of HNSCC of young and old patients

Author	Age	No. of patients	Years	Sex(M/F)	The most common site	The most common Stage	Grade	Country
Chidzonga et al. [32]	≤40 >40	69(19.3%) 289(80.7%)	1982-1991	40/29 200/89	F/T F/T		W W	Zimbabwe
Samreen et al. [33]	≤40 >40	19(21.8%) 68(78.2%)	2010-2013		OC OC	II II		Pakistan
Farnaz et al. [34]	20-40	21(13.3%)	1996-2009	12/9	T	I		Iran
Negar et al. [30]	≤40 >40	15(5.7%) 247(94.3)	1995-2010	9/6 210/27	T/L L	IV I	W W	Iran
Fan et al. [35]	22-44	100	2001-2010	66/34	T	II	W	*China*
Chang et al. [36]	<45 ≥45	608(25.9%) 1731(74.1%)	2004-2005	591/17 1628/103	B B			Taiwan
Iamaroon et al. [37]	≤45 >45	75 (12.8%) 512(87.2%)	1991-2000	43/32 294/218	T T	IV IV	W W	Thailand
Sasaki et al. [3]	≤40 >40	35(6.6%) 494(93.4)	1990-1999	20/15	T	I	W	UK
Ho et al. [38]	27-45	28	1999-2005	27/1	B	I/II		Taiwan
Lype et al. [25]	<35	115	1982-1996	74/41	T	III/IV	W	India
Pitman et al. [29]	16-39	122	1982-1994	70/52	T	I/II		US
Regan et al. [39]	≤40 >40	20(23.1%) 100(76.9%)	1993-2003	19/11	T Ph	I III	M M	Ireland

T=Toung, OC = oral cavity, Ph = Pharynx, L = Larynx, B=Buccal mucosa, F= Floor of the mouth.

Grade (M: Moderate, W: Well differentiated)

A crowd of previous reports concluded that HNSCC was more aggressive and the prognosis was poorer in young adults compared with the older patients [40–43]. Kuriakose et al. found that the tumors of young patients manifest predominantly as invasive lesions with early spread to lymph nodes. However, in the old patients, tumors occur more frequently in social classes III and IV and have association with smoking, alcohol or pan chewing, present as exophytic lesions and spread late to lymph nodes [44]. Another reason may be due to different degree of preventive system, the quality of health care and the attitudes of young patients towards visiting a doctor. The disease is often more difficult to diagnose pathologically and even misdiagnosed, and appropriate treatment is delayed when the case present. In clinical practice, HNSCC in young patients seems to be frequently diagnosed in its advanced stages [45], which leads to delay of rational therapy and may finally result in poorer prognosis for the patients [46]. However, there are still some recent researchers frown on this opinion, they hold that the comorbidities on patients of head and neck cancer affects the older patients more [47], and the older patients often die earlier compared to those who are free of diseases apart from HNSCC [48].

**Figure 1:**
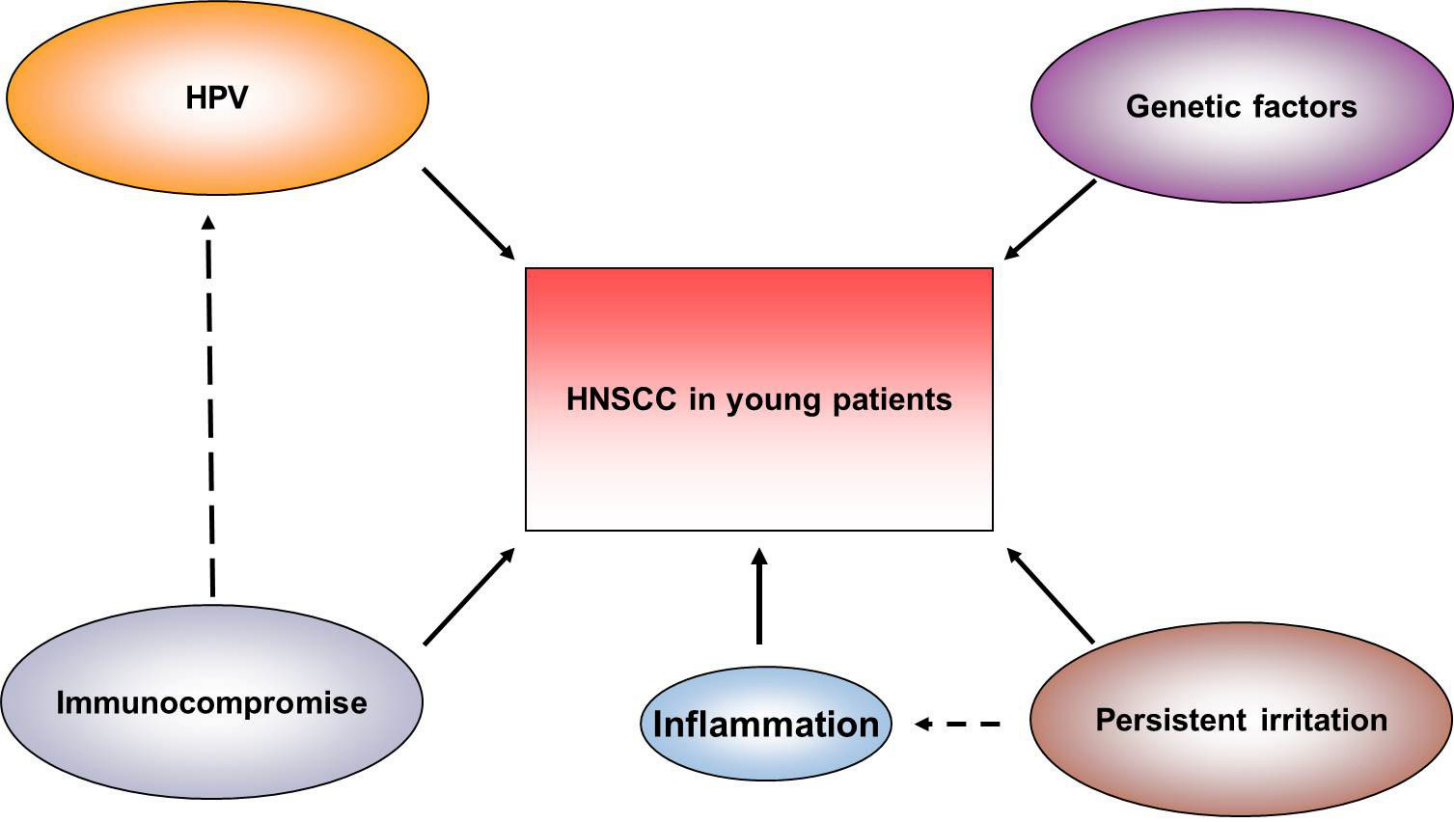
Risk factors of HNSCC in young patients

Risk factors for the increased frequency of young-onset HNSCC remain ill defined [49]. Strangely, most of the young patients are lack of significant exposure to alcohol and tobacco, this difference makes the etiology of young-onset HNSCC a hot topic, and it's necessary to get a deeper understanding of its pathogenesis in order to promote the development of improved therapeutic approaches. Studies show that part of HNSCC in young population appears to be due to rising numbers of HPV infection, particularly in males (smokers and non-smokers). There also exist other risk factors like persistent irritation in oral cavity, systemic syndromes, and immunosuppression in young patients with HNSCC. These special risk factors make young-onset HNSCC in young patients may result in its different hallmarks from the old patients. Nevertheless, the etiologic factors in young individuals with HNSCC under 45 years old remain unknown. In this review, we would like to highlight the special etiological spectrum of HNSCC in this population.

## HUMAN PAPILLOMAVIRUS (HPV)

Experimental models and analyses hold that viral infections may be an etiologic factor of oral cancers [50]. HPV is a well-established initiator of both cervical and anogenital carcinogenesis, and HPV DNA is found in nearly 100% of cervical carcinomas and 84% of anal carcinomas [51]. Recently, the rate of human papillomavirus (HPV)-induced head and neck squamous cell carcinomas (HNSCCs) seems to have increased, and especially, exposure to HPV has been identified as a major contributor to the pathogenesis of oropharyngeal carcinomas in young patients [5]. Kreimer et al. analyzed the data from 60 studies done in 26 countries, a significantly higher percentage of HPV infection was found in OPSCC (34 %) than in oral and laryngeal cancers (both 25 %) [52]. From 1973 to 2004, the rate of HPV-related OPSCC in the United States has increased obviously, especially in tonsillar [15, 53]. In Australia, the incidence of HPV-related sites in the oropharynx in men and women increased by 1% per year in the period 1982-2005 [54]. Hammarstedt et al. found that from 1970 to 2002, a nearly three-fold increase in the proportion of HPV-positive tonsillar cancer cases in Stockholm, Sweden was observed, and the distribution of HPV-positive cases was 23.3% in the 1970s, 29% in the 1980s, 57% in the 1990s and 68% during 2000-2002 [55].

HPV is an epitheliotropic, double stranded, circular DNA virus from papovavirus family, more than 150 different HPV subtypes identified to date and it is divided into low-risk and high-risk (HR) types [56]. Infection with high-risk types of HPV has been increasingly recognized as an important risk factor of carcinogenesis [57]. HPV oncogenic protein E6 and E7 lead to disruption of key tumor suppressor genes p53 and Rb, respectively, bringing about cell cycle deregulation and genomic instability [58–60]. High-risk type of HPV(especially HPV16) DNA has been detected in about 20-25% of HNSCC and up to 60% of the OPSCC [5, 52, 61]. Compared to HPV-negative OPSCC, HPV-positive tumors are characterized by the absence of P53 mutation [62, 63] and a lower number of chromosomal abnormalities [64]. Rb degradation also induces overexpression of P16, as a tumor suppressor protein, a hallmark of HPV-16-positive cancer of the oropharynx [54, 65].

Demographically, patients with HPV-positive HNSCC are more inclined to be youngsters, male, nonsmokers, nondrinkers and often have a higher socieconomic status [15, 66–68]. HPV is the most common sexually transmitted infection in adults, however, there existing several potential modes of the transmission of HPV to youngsters, like indirect transmission via fomites and perinatal vertical transmission, auto-and hetero-inoculation, and sexual ways such as sexual abuse [69, 70]. About vertical transmission, some researchers harbor the idea that there is very low risk for children of women with cervical cancer(women who have been infected by HR-HPV before pregnancy), to acquire and sustain HR-HPV in their oral cavity until childhood or adolescence [56], while other researchers hold that in the paediatric age infection is mostly connected with mother-to-child transmission, generally via the delivery duct [71]. Hence, for more accurate evaluation of vertical transmission, long term follow-up studies should be conducted. In addition, oral sex is supposed to be the most unnatural sex which is commonly practiced across the world and is proved to be a potential transmitting link between cancers of the oropharynx [72]. As it is perceived to be a form of safe sex, oral sex is widely accepted among teenagers and adults which results in an increased prevalence of HPV infection in young population [73]. Moreover, Espada et al. reported that that average age of sexual behavior has decreased, and simultaneously, the number of sexual partners has increased these years [74]. Hence, a history of genital warts, the number of sexual partners and young age are related to the transmission of HPV [75], HPV can also occur through contact with hands, feet and mouth, even a slight injury in the mucous membrane serves as an entry gate for HPV, which thus works into the basal layer of the epithelium [76, 77].

Patients with HPV associated oral cancer have a significantly better survival rate, than patients with HPV-negative cancer [51], irrespective of the treatment modality [50]. The higher survival rate among HPV-positive patients is in part attribute to greater locoregional control, higher sensitivity to radiation or better radiosensitization by Cisplatin-based chemoradiation strategies [78]. As p53 tumor-suppressor gene is often mutated in HPV-negative cancers, in HPV-positive cells, there existing intact p53 , making them sensitive to treatment and restore apoptotic function [79]. A study reported that far fewer genes were mutated per tumor in the HPV-associated tumors(4.8±3) as compared with those tumors not epidemiologically related to HPV (versus 20.6 ±16.7) [80]. Nicolas et al. reported that the Mutational Landscape of HNSCC and reported that the mutation rate of HPV-positive tumors was approximately half of that found in HPV-negative HNSCC (2.28 mutations per megabase as compared with 4.83 mutations per megabase), and it's suggestive of biological differences between HPV-positive and -negative disease [81]. Aberrant DNA hypermethylation within gene promoters is a hallmark in various human malignancies, including HNSCC. Efterpi et al. did an analysis based on the robust difference in HPV-dependent gene promoter hypermethylation in 3 OPSCC patient cohorts , and they identified that a combined promoter methylation pattern of low methylation levels in ALDH1A2 and OSR2 promoters and high methylation levels in GATA4, GRIA4, and IRX4 promoters was significantly correlated with better OPSCC outcome survival in 3 independent patient cohorts [82]. In addition, EGFR protein overexpression is reported in 70-90% of HNSCC in various studies [83] and has been correlated with a worse prognosis and resistance to radiation therapy in HNSCC [84, 85].

Furthermore, despite chronic HPV infection allows for the evolution of immune evasion mechanisms, and that inflammation associated to the oral cavity has been shown to be tumor promoting, viral antigens can elicit an immune response, and among HPV-positive patients, immune infiltration is associated with better prognosis [86–88]. The epithelium of the oropharyngeal differs from the mucosal epithelium at other HPV16-infected sites , and it is juxtaposed with the underlying lymphatic tissue, by which inhaled and ingested pathogens are surveilled, thus, the immune response to HPV at this site may be different from that at other anatomic locations [89]. HPV16-specific CD4 +and CD8+T-cells are more common in peripheral blood from HPV-positive HNSCC patients than HPV-negative HNSCC patients [90]. And the interplay between CD8+ and CD4+ T-cells can locally and systemically influence prognosis. Lin et al. showed that HPV-specific CD4+ T cells is of vital importance in the generation of HPV-specific CD8+ T cells, and that the CD4+ response plays a critical role in long-term tumor protection [91]. Nasman et al. found a significantly higher number of CD8+ tumor infiltrating lymphocytes and Foxp3+ Tregs in HPV-positive tonsillar SCC patients as compared with HPV-negative patients, and higher level of CD8+ T-cell infiltration and a high local CD8+ /Treg ratio were associated with better clinical outcome in tonsillar SCC cases [92]. Therefore, the immunologic profile of HPV-positive OPSCC plays a role in the significantly better outcome. Additionally, the HPV-specific immune response is reported to be associated with better response to therapies. Higher CD8 levels were also associated with tumor response to induction chemotherapy. Derrick and Hoffmann found a significantly higher levels of CD8+ T lymphocytes that are specific for HPV16 E7 peptides in the peripheral blood of HPV-positive patients, which lead to a better survival rate of HPV-positive patients after chemotherapy [93, 94]. Moreover, the tumor microenvironment, including immune cells, may contribute to the greater radiosensitivity , and the administration of radiation might enhance immune surveillance to viral antigens [86]. The mechanisms underlying HPV interferes with immune surveillance include: 1) Hindering Langerhans cell migration [95, 96] and activation [97], 2)Suppressing the interferon (IFN) response [98], 3) Interfering with HLA class 1-mediated antigen presentation [99], 4) suppression of inhibitory signals by T regulatory cells (Treg) and immature myeloid cells. However, the molecular mechanisms responsible for the better prognosis of HPV-positive HNSCC patients needed to be further explored.

## GENETIC FACTORS

Genetic predisposition may act as an important contributor to young-onset HSNCC [5]. The development of typical HNSCC is a result of a multistep process, which involves multiple genetic and epigenetic events . Genetic instability, as an important molecular mechanism in cancers of head and neck region, has been extensively studied .There exists a high consistency in relation to copy number gains and losses of specific patterns of DNA. Studies show that gains at 3q, 5q, 8q, and 11q, as well as losses at 3 and 9p have been found in most of the HNSCC patients [100]. Loss of chromosome region 3p and 9p21 are common early genetic events in head and neck squamous neoplasia [101, 102]. A study of young-onset HNSCC compared young adults (<40, *N* = 10) with mostly smoking older adults(≥40, *N* = 10) using array comparative genomic hybridization, They found that the meannumber of aberrations of young nonsmokers was less than 50% of that in the older smokers, which suggested that the genetic alterations of the young cohort was not seen so consistently in older HNSCC [103]. 3p losses, a frequent event in the old cohort , was not commonly found(only two from ten) in the young cohort . As to 9p21, it was striking that all of the young-onset HNSCCs lacked losses in this region, while in the older cases of HNSCC, this altered region consistently existed. 11q13 is a region that contains several genes(CCDN1, EMS 1, FGF3 and FGF4) previously shown to be amplified and overexpressed in HNSCC patients, while studies concluded that 11q13 amplification was significantly more frequent in smokers, which suggested that gains in this region may have no relationship with ages of patients [104].

EGFR is an important therapeutic target and prognostic factor in oral cancer. Overexpression of EGFR, results from regulatory pathway changes, gene structure changes or gene amplification, which plays a rather important role in tumorigenesis. Studies have shown that a high EGFR gene copy number was significantly more common in nonsmokers, as were EGFR mutations [105].

p53 gene alterations have been documented as the most frequent genetic event in HNSCC and cases with p53 alteration account for over 70% of the whole [106]. Previous studies show that overexpression of p53 in HNSCC in young nonsmokers is not associated with the classic p53 mutations in exons 5-9 [104]. And it has been shown that p53 sequence alterations are decreased in HPV infected patients, with the p53 silencing with its production of E6 [50].

p16, encoded by the CDKN2A gene locus in chromosome 9p21, is frequently inactivated resulting in a complete block of gene transcription in cancer patients [107]. In HNSCC patients, p16 is predominantly inactivated by methylation and deletion of the gene but not by mutation, and p16 methylation is a more common event in those patients under 40 years of age in contrast to p16 deletions, which are more generally seen in those older than 40 years. It appears that specific ways of inactivation of p16 in cancer of head and neck are related to specific patient risk profiles(p16INK4A genetic and epigenetic profiles differ in relation to age and site in head and neck squamous cell carcinomas). On the other hand, p16 overexpression has also been found in HNSCC patients with HPV infection(we have discussed it in the part of HPV infection) [108].

Moreover, Several SNPs may attenuate the function of specific proteins and are associated with increased risk of several malignancies, and such SNPs affected genes involved in repair of DNA damage induced by tobacco, and prominent cell cycle regulators like Cyclin D1 (CCND1), p53 and P21 (Waf1/CIP1) [5]. For example, the recently identified single nucleotide polymorphism in the MDM2 promoter (SNP 309) may contribute to the early onset of in patients of HNSCC with defective p53. Huang et al. reported that the mean age of onset for male OSCC patients with the MDM2 SNP 309 GG genotype was 4.45 years earlier than those with the MDM2 SNP 309 TT genotype (*p* = 0.02) and with poorer differentiation of OSCC tumors (*p* = 0.01) [112] .

Patients having one of several well-defined inherited syndromes like Fanconi's anaemia, Epidermolysis bullosa, Xeroderma pigmentosum, and Blooms syndrome feature a significantly increased incidence of HNSCC estimated 700-1000 fold greater than that of general people [109]. Such patients have Increased susceptibility to viral transformation, susceptibility to mutagens, possible immune system defects, and especially chromosomal instability and defective DNA repair [110]. Various organs can be affected and individuals generally present with complications such as growth retardation, congenital malformations, hyper-pigmentation and an elevated risk of secondary malignancies including HNSCC at a early age [111]. It's worth noting that sporadic HNSCC that develop in the general population has relationship with inactivation of these genes (TP53, p16, FANCA-M) or aberrant expression of the members of these oncogenes (ras and myc gene family, int-2, hst-1, cyclin D1, epidermal growth factor receptor and Bcl-2) [112]. Similarly, pathways involved in surveillance of genetic stability are pivotal events in HNSCC development in individuals with such HNSCC susceptibility syndromes. Although without enough evidence, researchers strongly highlighted the significance to carry out an objective, thorough and standardised oral cavity examination and genetic consultation of young population with such systemic diseases [71].

Additionally, family history may open up important clues for HNSCC susceptibility, and it has a relationship with an earlier onset of HNSCC [5]. People who have a family history of one first-degree relative with the same tumor and the same clinical features, with two or more first-degree relatives with tumors at the same site or with two or more first-degree relatives with rare tumors are strongly suspect for an underlying genetic disorder [113]. Individuals having a family history of cancer in three or more first-degree relatives have a 2-fold increased risk of HNSCC [114]. The genetic law of cancer is quite special, the vast majority of cancers can be explained by “inherited susceptibility”, the offspring do not inherit cancer itself but a kind of individual quality which makes them easier to get cancer [115, 116].

## IMMUNOCOMPROMISE

The concept of immune surveillance was first put forward by Burnett, that the immune system has monitor functions of tumor, so that new malignant transformation cells can be removed at any time preventing tumors from happening [117, 118]. Researches show that T cell deficiency or disruption of specific cytotoxic mechanisms can render experimental animals more susceptible to spontaneous or chemical carcinogenesis [119, 120]. CD4+T cell deficiency and function disruption are hallmarks of acquired immunodeficiency syndrome (AIDS), caused by the human immunodeficiency virus (HIV) [119, 120]. The risks of several different cancer types such as lung cancer, esophagus cancer and breast cancer are elevated in HIV-infected individuals due to immunodeficiency or immune dysfunction/senescence [121, 122]. Large cohort and HIV/AIDS-Cancer registry match studies have suggested that HIV-infected individuals have a 1.5-4-fold higher risk for HNSCC comparing with the general population [123, 124]. Furthermore, HIV-related immunosuppression may be a strong risk factor for oral HPV incidence or persistence given the 2-3 times higher adjusted odds of oral HPV prevalence in HIV-infected individuals compared to HIV-uninfected individuals [125]. And recent studies suggested HIV-infected individuals have an overall oral HPV DNA prevalence between 20% and 45%, and an oncogenic oral HPV DNA prevalence between 12% and 26% [125–127]. And the increased oral HPV prevalence may reflect a loss of vital control in those with compromised immune system inversely. Considering special ages of HIV and HPV infection, it's reasonable to speculate that head and neck squamous cell carcinoma related to AIDS might accordingly occur in the young population.

Patients who have received solid organ transplantation (SOT) have an increased risk of suffering from malignancies [128], and the development of malignancy after SOT may be explained by the role of the immune system , infections, and some other factors [129]. Many studies show that the overall incidence of HNSCC is increased in recipients especially liver recipients who had alcoholic cirrhosis compared with the general population [130]. And it's reported that about 4-15% of all malignant tumors after transplantation are head and neck cancers and a large part of these head and neck cancers are squamous cell carcinomas of the lip or oropharynx [128, 130–134]. Jenny et al reported that SOT patients have the risk increased 6 times for oral cancer, and for lip cancer 44 times in their cohort, and SOT patients with lip cancer have a worse prognosis compared with non-SOT patients [135]. In addition, Head and neck malignancies after SOT are inclined to affect younger recipients, and often have a more aggressive behavior, a worse prognosis [136, 137]. Annekatrin et al. reported that 33 out of 2040 liver recipients developed de novo head and neck malignancies, and the median age at liver transplantation was 51 years (17-64 years) and the median time of diagnosis of HNSCC after transplantation was 7 years (1-25 years), it suggested that solid organ transplantation is a possible risk factor of young-onset HNSCC [138].

## PERSISTENT IRRITATION

Persistent irritation on oral mucosa was reported to have potential to trigger chronic inflammation, giving rise to the occurrence of oral cancer. Irritations constantly do harm to the epithelia, which may reduce their barrier function and make them more vulnerable for pathogens. On the other hand, to supersede damaged cells and repair mucosal lesions, higher mitosis efficiency and proliferation are necessary, which may bring about an increased vulnerability, genetic instability and accumulation of somatic mutations. The latter leads to increased risk of oral cancer [57]. Santos-Silva et al. reported that 3 young patients (between 21 to 34 years of age) , who had recently received orthodontic treatment , were diagnosed with tongue squamous cell carcinoma subsequently [139]. Consistent irritation in oral cavity is also visible in children, which may increase the risk of them to suffer from OSCC. Moore reported a case of tongue carcinoma induced by the spur of a nonviable broken tooth in a 13-year-old boy [140]. A case reported by Amichetti of a 14-year-old girl used to chewing a plastic ball pen finally suffered from OSCC [141]. Another patient at age of 14 encountered OSCC after the stimulation of orthodontic appliance [142].

Recent studies have shown that in addition to being a tumor initiator by virtue of its high carcinogen content, tobacco and alcohol use are also tumor promoters because of their ability to trigger chronic inflammation [143]. Such commonly accepted risk factors, which involved in the genesis of carcinoma in adulthood, have little association with HNSCC in paediatric patients and adolescents, this is because of the fact that a very short-term exposure is not enough to induce neoplastic degeneration [144, 145]. However, based on the far-ranging tobacco control activities, the number of adult smokers decreased evidently, while the number of smoking in young population especially in developing countries increased significantly in these years. Data from Eurobarometer in 2009 showed that 46 % of men and 32 % of women smoked more than 15 regularly. 39 % in 15-24 age group, and 50 % in 25-39 age group were smokers [51]. Such high number of smokers in the young population should not be ignored. Wechsler et al. reported that an increasing frequency of binge drinking behavior among teenagers became a major concern area of young-age alcohol consumption. In the US, data from National Household Survey showed that about half of the young drinkers(18-25 years old) were binge drinkers and about one in five were heavy drinkers [146]. The Global Status Report “Alcohol and young people” reports that young people are starting to drink at earlier ages [5]. Thus, regular alcohol intake in Western youth is decreasing while heavy consumption of alcohol over a short period of time (binge drinking) has become more popular in recent years. Strangely, a large number of patients especially most young women patients, who don't even have a habit of smoking or drinking, they still suffer from HNSCC, it may result from variation in ability to detoxify the smoke and alcohol products in different individuals, therefore, environmental exposure to smoke may play a role in such none-smokers with HNSCC [147].

In addition, poor oral health can easily lead to mucosal disruption by local irritation, patients have increased susceptibility to infectiousness of HPV and oral cancer [148]. Wolfgang et al. harbored that both poor oral health and poor dental care were positively associated with increased risk of oral and oropharyngeal cancers, which could not be explained by smoking, alcohol or other known risk factors [149]. Children may be easier to get poor oral health because most of them lack the awareness of oral hygiene such as irregular tooth brushing, too much sugar or sugar-containing foods consumption. Especially in developing countries, education of the importance of oral health lags behind relatively, parents are lack of knowledge, which affects children oral health seriously [150]. Thus, the improvements in the level of knowledge and awareness of school health care instructor is necessary for the children.

## CONCLUSIONS

Young-onset HNSCC, though uncommon, the number of which is increasing at a great lick, In addition to the traditional risk factors like tobacco and alcohol using, head and neck cancer in young patients may also be associated with viral infection, genetic factors, immune factors and persistent irritation, etc. While a very short-term exposure may not be enough to induce tumorigenesis, and studies found significant differences in gene and prognosis between young and old patients, which indicate that young-onset HNSCC may be a new kind of disease apart from HNSCC in old patients. Thus, improved definition of this biology is needed to establish successful preventive and treatment efforts for HNSCC in young patients.

## References

[1] Parkin DM, Bray F, Ferlay J, Pisani P (2005). Global cancer statistics, 2002. CA Cancer J Clin.

[2] van Monsjou HS, Balm AJ, van den Brekel MM, Wreesmann VB (2010). Oropharyngeal squamous cell carcinoma: a unique disease on the rise?. Oral Oncol.

[3] Sasaki T, Moles DR, Imai Y, Speight PM (2005). Clinico-pathological features of squamous cell carcinoma of the oral cavity in patients <40 years of age. J Oral Path Med.

[4] Chitapanarux I, Lorvidhaya V, Sittitrai P, Pattarasakulchai T, Tharavichitkul E, Sriuthaisiriwong P, Kamnerdsupaphon P, Sukthomya V (2006). Oral cavity cancers at a young age: analysis of patient, tumor and treatment characteristics in Chiang Mai University Hospital. Oral Oncol.

[5] van Monsjou HS, Wreesmann VB, van den Brekel MW, Balm AJ (2013). Head and neck squamous cell carcinoma in young patients. Oral Oncol.

[6] Hart AK, Karakla DW, Pitman KT, Adams JF (1999). Oral and oropharyngeal squamous cell carcinoma in young adults: a report on 13 cases and review of the literature. Otolaryngol Head Neck Surg.

[7] Shiboski CH, Schmidt BL, Jordan RC (2005). Tongue and tonsil carcinoma: increasing trends in the U.S. population ages 20-44 years. Cancer.

[8] Annertz K, Anderson H, Biorklund A, Moller T, Kantola S, Mork J, Olsen JH, Wennerberg J (2002). Incidence and survival of squamous cell carcinoma of the tongue in Scandinavia, with special reference to young adults. Int J Cancer.

[9] Patel SC, Carpenter WR, Tyree S, Couch ME, Weissler M, Hackman T, Hayes DN, Shores C, Chera BS (2011). Increasing incidence of oral tongue squamous cell carcinoma in young white women, age 18 to 44 years. J Clin Oncol.

[10] Cusumano RJ, Persky MS (1988). Squamous cell carcinoma of the oral cavity and oropharynx in young adults. Head Neck Surg.

[11] Llewellyn CD, Johnson NW, Warnakulasuriya KA (2001). Risk factors for squamous cell carcinoma of the oral cavity in young people—a comprehensive literature review. Oral Oncol.

[12] Nair U, Bartsch H, Nair J (2004). Alert for an epidemic of oral cancer due to use of the betel quid substitutes gutkha and pan masala: a review of agents and causative mechanisms. Mutagenesis.

[13] Gupta PC (1999). Mouth cancer in India: a new epidemic?. J Indian Med Assoc.

[14] Chen K, Song F, He M, Li H, Qian B, Zhang W, Wei Q, Hao X (2009). Trends in head and neck cancer incidence in Tianjin, China, between 1981 and 2002. Head Neck.

[15] Chaturvedi AK, Engels EA, Anderson WF, Gillison ML (2008). Incidence trends for human papillomavirus-related and -unrelated oral squamous cell carcinomas in the United States. J Clin Oncol.

[16] Conway DI, Stockton DL, Warnakulasuriya KA, Ogden G, Macpherson LM (2006). Incidence of oral and oropharyngeal cancer in United Kingdom (1990-1999) — recent trends and regional variation. Oral Oncol.

[17] Schantz SP, Yu GP (2002). Head and neck cancer incidence trends in young Americans, 1973-1997, with a special analysis for tongue cancer. Arch Otolaryngol Head Neck Surg.

[18] Lacy PD, Piccirillo JF, Merritt MG, Zequeira MR (2000). Head and neck squamous cell carcinoma: better to be young. Otolaryngol Head Neck Surg.

[19] Doobaree IU, Landis SH, Linklater KM, El-Hariry I, Moller H, Tyczynski J (2009). Head and neck cancer in South East England between 1995-1999 and 2000-2004: An estimation of incidence and distribution by site, stage and histological type. Oral Oncol.

[20] Muller S, Pan Y, Li R, Chi AC (2008). Changing trends in oral squamous cell carcinoma with particular reference to young patients: 1971-2006. The Emory University experience. Head Neck Pathol.

[21] Braakhuis BJ, Visser O, Leemans CR (2009). Oral and oropharyngeal cancer in The Netherlands between 1989 and 2006: Increasing incidence, but not in young adults. Oral Oncol.

[22] Davis S, Severson RK (1987). Increasing incidence of cancer of the tongue in the United States among young adults. Lancet.

[23] Macfarlane GJ, Boyle P, Scully C (1992). Oral cancer in Scotland: changing incidence and mortality. BMJ.

[24] Funk GF, Karnell LH, Robinson RA, Zhen WK, Trask DK, Hoffman HT (2002). Presentation, treatment, and outcome of oral cavity cancer: a National Cancer Data Base report. Head Neck.

[25] Iype EM, Pandey M, Mathew A, Thomas G, Sebastian P, Nair MK (2001). Oral cancer among patients under the age of 35 years. J Postgrad Med.

[26] Llewellyn CD, Linklater K, Bell J, Johnson NW, Warnakulasuriya KA (2003). Squamous cell carcinoma of the oral cavity in patients aged 45 years and under: a descriptive analysis of 116 cases diagnosed in the South East of England from 1990 to 1997. Oral Oncol.

[27] Veness MJ, Morgan GJ, Sathiyaseelan Y, Gebski V (2003). Anterior tongue cancer: age is not a predictor of outcome and should not alter treatment. Anz J Surg.

[28] Verschuur HP, Irish JC, O'sullivan B, Goh C, Gullane PJ, Pintilie M (1999). A matched control study of treatment outcome in young patients with squamous cell carcinoma of the head and neck. LARYNGOSCOPE.

[29] Pitman KT, Johnson JT, Wagner RL, Myers EN (2000). Cancer of the tongue in patients less than forty. Head Neck.

[30] Mafi N, Kadivar M, Hosseini N, Ahmadi S, Zare-Mirzaie A (2012). Head and neck squamous cell carcinoma in Iranian patients and risk factors in young adults: a fifteen-year study. Asian Pac J Cancer Prev.

[31] Harris SL, Kimple RJ, Hayes DN, Couch ME, Rosenman JG (2010). Never-smokers, never-drinkers: unique clinical subgroup of young patients with head and neck squamous cell cancers. Head Neck.

[32] Chidzonga MM, Mahomva L (2006). Squamous cell carcinoma of the oral cavity, maxillary antrum and lip in a Zimbabwean population: a descriptive epidemiological study. Oral Oncol.

[33] Naz S, Salah K, Khurshid A, Hashmi AA, Faridi N (2015). Head and neck squamous cell carcinoma - comparative evaluation of pathological parameters in young and old patients. Asian Pac J Cancer Prev.

[34] Falaki F, Dalirsani Z, Pakfetrat A, Falaki A, Saghravanian N, Nosratzehi T, Pazouki M (2011). Clinical and histopathological analysis of oral squamous cell carcinoma of young patients in Mashhad, Iran: a retrospective study and review of literature. Med Oral Patol Oral Cir Bucal.

[35] Fan Y, Zheng L, Mao MH, Huang MW, Liu SM, Zhang J, Li SL, Zheng L, Zhang JG (2014). Survival analysis of oral squamous cell carcinoma in a subgroup of young patients. Asian Pac J Cancer Prev.

[36] Chang TS, Chang CM, Ho HC, Su YC, Chen LF, Chou P, Lee CC (2013). Impact of young age on the prognosis for oral cancer: a population-based study in Taiwan. Plos One.

[37] Iamaroon A, Pattanaporn K, Pongsiriwet S, Wanachantararak S, Prapayasatok S, Jittidecharaks S, Chitapanarux I, Lorvidhaya V (2004). Analysis of 587 cases of oral squamous cell carcinoma in northern Thailand with a focus on young people. Int J Oral Maxillofac Surg.

[38] Ho HC, Lee MS, Hsiao SH, Hwang JH, Hung SK, Chou P, Lee CC (2008). Squamous cell carcinoma of the oral cavity in young patients: a matched-pair analysis. Eur Arch Otorhinolaryngol.

[39] O'Regan EM, Timon C, Sheils O, Codd M, O'Leary JJ, Toner M (2006). Squamous cell carcinoma of the head and neck in young Irish adults. Br J Oral Maxillofac Surg.

[40] Majchrzak E, Szybiak B, Wegner A, Pienkowski P, Pazdrowski J, Luczewski L, Sowka M, Golusinski P, Malicki J, Golusinski W (2014). Oral cavity and oropharyngeal squamous cell carcinoma in young adults: a review of the literature. Radiol Oncol.

[41] Amsterdam JT, Strawitz JG (1982). Squamous cell carcinoma of the oral cavity in young adults. J Surg Oncol.

[42] Sarkaria JN, Harari PM (1994). Oral tongue cancer in young adults less than 40 years of age: rationale for aggressive therapy. Head Neck.

[43] Son YH, Kapp DS (1985). Oral cavity and oropharyngeal cancer in a younger population. Review of literature and experience at Yale. Cancer.

[44] Kuriakose M, Sankaranarayanan M, Nair MK, Cherian T, Sugar AW, Scully C, Prime SS (1992). Comparison of oral squamous cell carcinoma in younger and older patients in India. Eur J Cancer B Oral Oncol.

[45] Ribeiro AC, Silva AR, Simonato LE, Salzedas LM, Sundefeld ML, Soubhia AM (2009). Clinical and histopathological analysis of oral squamous cell carcinoma in young people: a descriptive study in Brazilians. Br J Oral Maxillofac Surg.

[46] Oliver RJ, Dearing J, Hindle I (2000). Oral cancer in young adults: report of three cases and review of the literature. Br Dent J.

[47] Sanabria A, Carvalho AL, Vartanian JG, Magrin J, Ikeda MK, Kowalski LP (2007). Comorbidity is a prognostic factor in elderly patients with head and neck cancer. Ann Surg Oncol.

[48] Paleri V, Wight RG, Silver CE, Haigentz MJ, Takes RP, Bradley PJ, Rinaldo A, Sanabria A, Bien S, Ferlito A (2010). Comorbidity in head and neck cancer: a critical appraisal and recommendations for practice. Oral Oncol.

[49] Gillison ML, Lowy DR (2004). A causal role for human papillomavirus in head and neck cancer. Lancet.

[50] Gillison ML, Koch WM, Capone RB, Spafford M, Westra WH, Wu L, Zahurak ML, Daniel RW, Viglione M, Symer DE, Shah KV, Sidransky D (2000). Evidence for a causal association between human papillomavirus and a subset of head and neck cancers. J Natl Cancer Inst.

[51] Turi K, Barabas P, Csurgay K, Lehner GY, Lorincz A, Nemeth ZS (2013). An analysis of the epidemiological and etiological factors of oral tumors of young adults in a Central-Eastern European population. Pathol Oncol Res.

[52] Kreimer AR, Clifford GM, Boyle P, Franceschi S (2005). Human papillomavirus types in head and neck squamous cell carcinomas worldwide: a systematic review. Cancer Epidemiol Biomarkers Prev.

[53] Ryerson AB, Peters ES, Coughlin SS, Chen VW, Gillison ML, Reichman ME, Wu X, Chaturvedi AK, Kawaoka K (2008). Burden of potentially human papillomavirus-associated cancers of the oropharynx and oral cavity in the US, 1998-2003. Cancer.

[54] Grulich AE, Jin F, Conway EL, Stein AN, Hocking J (2010). Cancers attributable to human papillomavirus infection. Sex Health.

[55] Hammarstedt L, Lindquist D, Dahlstrand H, Romanitan M, Dahlgren LO, Joneberg J, Creson N, Lindholm J, Ye W, Dalianis T, Munck-Wikland E (2006). Human papillomavirus as a risk factor for the increase in incidence of tonsillar cancer. IntJ Cancer.

[56] Saini R, Khim TP, Rahman SA, Ismail M, Tang TH (2010). High-risk human papillomavirus in the oral cavity of women with cervical cancer, and their children. Virol J.

[57] Dietz A, Wichmann G (2011). Head and neck cancer: effective prevention in youth and predictive diagnostics for personalised treatment strategies according to biological differences. Epma J.

[58] Pytynia KB, Dahlstrom KR, Sturgis EM (2014). Epidemiology of HPV-associated oropharyngeal cancer. Oral Oncol.

[59] Zur HH (2002). Papillomaviruses and cancer: from basic studies to clinical application. Nat Rev Cancer.

[60] Rampias T, Sasaki C, Weinberger P, Psyrri A (2009). E6 and e7 gene silencing and transformed phenotype of human papillomavirus 16-positive oropharyngeal cancer cells. J Natl Cancer Inst.

[61] Ernster JA, Sciotto CG, O'Brien MM, Finch JL, Robinson LJ, Willson T, Mathews M (2007). Rising incidence of oropharyngeal cancer and the role of oncogenic human papilloma virus. Laryngoscope.

[62] Shi W, Kato H, Perez-Ordonez B, Pintilie M, Huang S, Hui A, O'sullivan B, Waldron J, Cummings B, Kim J, Ringash J, Dawson LA, Gullane P, Siu L, Gillison M, Liu FF (2009). Comparative prognostic value of HPV16 E6 mRNA compared with in situ hybridization for human oropharyngeal squamous carcinoma. J Clin Oncol.

[63] van Monsjou HS, van Velthuysen ML, van den Brekel MW, Jordanova ES, Melief CJ, Balm AJ (2012). Human papillomavirus status in young patients with head and neck squamous cell carcinoma. Int J Cancer.

[64] Smeets SJ, Braakhuis BJ, Abbas S, Snijders PJ, Ylstra B, van de Wiel MA, Meijer GA, Leemans CR, Brakenhoff RH (2006). Genome-wide DNA copy number alterations in head and neck squamous cell carcinomas with or without oncogene-expressing human papillomavirus. Oncogene.

[65] Weinberger PM, Yu Z, Haffty BG, Kowalski D, Harigopal M, Brandsma J, Sasaki C, Joe J, Camp RL, Rimm DL, Psyrri A (2006). Molecular classification identifies a subset of human papillomavirus—associated oropharyngeal cancers with favorable prognosis. J Clin Oncol.

[66] Ang KK, Harris J, Wheeler R, Weber R, Rosenthal DI, Nguyen-Tan PF, Westra WH, Chung CH, Jordan RC, Lu C, Kim H, Axelrod R, Silverman CC, Redmond KP, Gillison ML (2010). Human papillomavirus and survival of patients with oropharyngeal cancer. N Engl J Med.

[67] D'souza G, Kreimer AR, Viscidi R, Pawlita M, Fakhry C, Koch WM, Westra WH, Gillison ML (2007). Case-control study of human papillomavirus and oropharyngeal cancer. N Engl J Med.

[68] Fakhry C, Westra WH, Li S, Cmelak A, Ridge JA, Pinto H, Forastiere A, Gillison ML (2008). Improved survival of patients with human papillomavirus-positive head and neck squamous cell carcinoma in a prospective clinical trial. J Natl Cancer Inst.

[69] Syrjanen S, Puranen M (2000). Human papillomavirus infections in children: the potential role of maternal transmission. Crit Rev Oral Biol Med.

[70] Sinal SH, Woods CR (2005). Human papillomavirus infections of the genital and respiratory tracts in young children. Semin Pediatr Infect Dis.

[71] Tettamanti L, Caprioglio A, Tecco S, Barello G, Macchi A, Tagliabue A, Levrini L (2012). Oral Squamous Cell Carcinoma in the paediatric patient: a literature review. Eur J Paediatr Dent.

[72] Mishra A, Verma V (2015). Oral Sex and HPV: Population Based Indications. Indian J Otolaryngol Head Neck Surg.

[73] Chaudhary AK, Singh M, Sundaram S, Mehrotra R (2009). Role of human papillomavirus and its detection in potentially malignant and malignant head and neck lesions: updated review. Head Neck Oncol.

[74] Espada JP, Escribano S, Orgiles M, Morales A, Guillen-Riquelme A (2015). Sexual risk behaviors increasing among adolescents over time: comparison of two cohorts in Spain. Aids Care.

[75] Schwartz SM, Daling JR, Doody DR, Wipf GC, Carter JJ, Madeleine MM, Mao EJ, Fitzgibbons ED, Huang S, Beckmann AM, McDougall JK, Galloway DA (1998). Oral cancer risk in relation to sexual history and evidence of human papillomavirus infection. J Natl Cancer Inst.

[76] Michl P, Pazdera J, Prochazka M, Pink R, Stosova T (2010). Human papillomavirus in the etiology of head and neck carcinomas. Biomed Pap Med Fac Univ Palacky Olomouc Czech Repub.

[77] Vidal L, Gillison ML (2008). Human papillomavirus in HNSCC: recognition of a distinct disease type. Hematol Oncol Clin North Am.

[78] Lassen P, Eriksen JG, Hamilton-Dutoit S, Tramm T, Alsner J, Overgaard J (2009). Effect of HPV-associated p16INK4A expression on response to radiotherapy and survival in squamous cell carcinoma of the head and neck. J Clin Oncol.

[79] Ferris RL, Martinez I, Sirianni N, Wang J, Lopez-Albaitero A, Gollin SM, Johnson JT, Khan S (2005). Human papillomavirus-16 associated squamous cell carcinoma of the head and neck (SCCHN): a natural disease model provides insights into viral carcinogenesis. Eur J Cancer.

[80] Agrawal N, Frederick MJ, Pickering CR, Bettegowda C, Chang K, Li RJ, Fakhry C, Xie TX, Zhang J, Wang J, Zhang N, El-Naggar AK, Jasser SA, Weinstein JN, Trevino L, Drummond JA (2011). Exome sequencing of head and neck squamous cell carcinoma reveals inactivating mutations in NOTCH1. Science.

[81] Stransky N, Egloff AM, Tward AD, Kostic AD, Cibulskis K, Sivachenko A, Kryukov GV, Lawrence MS, Sougnez C, McKenna A, Shefler E, Ramos AH, Stojanov P, Carter SL, Voet D, Cortes ML (2011). The mutational landscape of head and neck squamous cell carcinoma. Science.

[82] Kostareli E, Holzinger D, Bogatyrova O, Hielscher T, Wichmann G, Keck M, Lahrmann B, Grabe N, Flechtenmacher C, Schmidt CR, Seiwert T, Dyckhoff G, Dietz A, Hofler D, Pawlita M, Benner A (2013). HPV-related methylation signature predicts survival in oropharyngeal squamous cell carcinomas. J Clin Invest.

[83] Roskoski RJ (2004). The ErbB/HER receptor protein-tyrosine kinases and cancer. Biochem Biophys Res Commun.

[84] Chung CH, Ely K, McGavran L, Varella-Garcia M, Parker J, Parker N, Jarrett C, Carter J, Murphy BA, Netterville J, Burkey BB, Sinard R, Cmelak A, Levy S, Yarbrough WG, Slebos RJ (2006). Increased epidermal growth factor receptor gene copy number is associated with poor prognosis in head and neck squamous cell carcinomas. J Clin Oncol.

[85] Zimmermann M, Zouhair A, Azria D, Ozsahin M (2006). The epidermal growth factor receptor (EGFR) in head and neck cancer: its role and treatment implications. Radiat Oncol.

[86] Vu HL, Sikora AG, Fu S, Kao J (2010). HPV-induced oropharyngeal cancer, immune response and response to therapy. Cancer Lett.

[87] Feller L, Altini M, Lemmer J (2013). Inflammation in the context of oral cancer. ORAL ONCOL.

[88] Andersen AS, Koldjaer SA, Ovesen T, Rusan M (2014). The interplay between HPV and host immunity in head and neck squamous cell carcinoma. Int J Cancer.

[89] Perry ME (1994). The specialised structure of crypt epithelium in the human palatine tonsil and its functional significance. J ANAT.

[90] Spanos WC, Nowicki P, Lee DW, Hoover A, Hostager B, Gupta A, Anderson ME, Lee JH (2009). Immune response during therapy with cisplatin or radiation for human papillomavirus-related head and neck cancer. Arch Otolaryngol Head Neck Surg.

[91] Lin CT, Chang TC, Shaw SW, Cheng PJ, Huang CT, Chao A, Soong YK, Lai CH (2006). Maintenance of CD8 effector T cells by CD4 helper T cells eradicates growing tumors and promotes long-term tumor immunity. Vaccine.

[92] Nasman A, Romanitan M, Nordfors C, Grun N, Johansson H, Hammarstedt L, Marklund L, Munck-Wikland E, Dalianis T, Ramqvist T (2012). Tumor infiltrating CD8+ and Foxp3+ lymphocytes correlate to clinical outcome and human papillomavirus (HPV) status in tonsillar cancer. Plos One.

[93] Wansom D, Light E, Worden F, Prince M, Urba S, Chepeha DB, Cordell K, Eisbruch A, Taylor J, D'silva N, Moyer J, Bradford CR, Kurnit D, Kumar B, Carey TE, Wolf GT (2010). Correlation of cellular immunity with human papillomavirus 16 status and outcome in patients with advanced oropharyngeal cancer. Arch Otolaryngol Head Neck Surg.

[94] Hoffmann TK, Arsov C, Schirlau K, Bas M, Friebe-Hoffmann U, Klussmann JP, Scheckenbach K, Balz V, Bier H, Whiteside TL (2006). T cells specific for HPV16 E7 epitopes in patients with squamous cell carcinoma of the oropharynx. Int J Cancer.

[95] Guess JC, McCance DJ (2005). Decreased migration of Langerhans precursor-like cells in response to human keratinocytes expressing human papillomavirus type 16 E6/E7 is related to reduced macrophage inflammatory protein-3alpha production. J Virol.

[96] Laurson J, Khan S, Chung R, Cross K, Raj K (2010). Epigenetic repression of E-cadherin by human papillomavirus 16 E7 protein. Carcinogenesis.

[97] Fausch SC, Fahey LM, Da SD, Kast WM (2005). Human papillomavirus can escape immune recognition through Langerhans cell phosphoinositide 3-kinase activation. J Immunol.

[98] Barnard P, Payne E, McMillan NA (2000). The human papillomavirus E7 protein is able to inhibit the antiviral and anti-growth functions of interferon-alpha. Virology.

[99] Ashrafi GH, Haghshenas MR, Marchetti B, O'Brien PM, Campo MS (2005). E5 protein of human papillomavirus type 16 selectively downregulates surface HLA class I. Int J Cancer.

[100] Hermsen MA, Joenje H, Arwert F, Braakhuis BJ, Baak JP, Westerveld A, Slater R (1997). Assessment of chromosomal gains and losses in oral squamous cell carcinoma by comparative genomic hybridisation. Oral Oncol.

[101] Califano J, van der Riet P, Westra W, Nawroz H, Clayman G, Piantadosi S, Corio R, Lee D, Greenberg B, Koch W, Sidransky D (1996). Genetic progression model for head and neck cancer: implications for field cancerization. Cancer Res.

[102] Garnis C, Baldwin C, Zhang L, Rosin MP, Lam WL (2003). Use of complete coverage array comparative genomic hybridization to define copy number alterations on chromosome 3p in oral squamous cell carcinomas. Cancer Res.

[103] Toner M, O'Regan EM (2009). Head and neck squamous cell carcinoma in the young: a spectrum or a distinct group? Part 2. Head Neck Pathol.

[104] Koch WM, Lango M, Sewell D, Zahurak M, Sidransky D (1999). Head and neck cancer in nonsmokers: a distinct clinical and molecular entity. Laryngoscope.

[105] Ryott M, Wangsa D, Heselmeyer-Haddad K, Lindholm J, Elmberger G, Auer G, Avall LE, Ried T, Munck-Wikland E (2009). EGFR protein overexpression and gene copy number increases in oral tongue squamous cell carcinoma. Eur J Cancer.

[106] De Paula AM, Souza LR, Farias LC, Correa GT, Fraga CA, Eleuterio NB, Silveira AC, Santos FB, Haikal DS, Guimaraes AL, Gomez RS (2009). Analysis of 724 cases of primary head and neck squamous cell carcinoma (HNSCC) with a focus on young patients and p53 immunolocalization. Oral Oncol.

[107] Reed AL, Califano J, Cairns P, Westra WH, Jones RM, Koch W, Ahrendt S, Eby Y, Sewell D, Nawroz H, Bartek J, Sidransky D (1996). High frequency of p16 (CDKN2/MTS-1/INK4A) inactivation in head and neck squamous cell carcinoma. Cancer Res.

[108] Mork J, Lie AK, Glattre E, Hallmans G, Jellum E, Koskela P, Moller B, Pukkala E, Schiller JT, Youngman L, Lehtinen M, Dillner J (2001). Human papillomavirus infection as a risk factor for squamous-cell carcinoma of the head and neck. N Engl J Med.

[109] Romick-Rosendale LE, Lui VW, Grandis JR, Wells SI (2013). The Fanconi anemia pathway: repairing the link between DNA damage and squamous cell carcinoma. Mutat Res.

[110] Budrukkar A, Shahid T, Murthy V, Hussain T, Mulherkar R, Vundinti BR, Deshpande M, Sengar M, Laskar SG, Agarwal JP (2010). Squamous cell carcinoma of base of tongue in a patient with Fanconi's anemia treated with radiation therapy: case report and review of literature. Head Neck.

[111] Kaplan MJ, Sabio H, Wanebo HJ, Cantrell RW (1985). Squamous cell carcinoma in the immunosuppressed patient: Fanconi's anemia. LARYNGOSCOPE.

[112] Huang SF, Chen IH, Liao CT, Wang HM, Liou SH, Hsieh LL (2009). Combined effects of MDM2 SNP 309 and p53 mutation on oral squamous cell carcinomas associated with areca quid chewing. Oral Oncol.

[113] Hill DA, Inskip PD, Shapiro WR, Selker RG, Fine HA, Black PM, Linet MS (2003). Cancer in first-degree relatives and risk of glioma in adults. Cancer Epidemiol Biomarkers Prev.

[114] Yu GP, Zhang ZF, Hsu TC, Spitz MR, Schantz SP (1999). Family history of cancer, mutagen sensitivity, and increased risk of head and neck cancer. Cancer Lett.

[115] Harty LC, Caporaso NE, Hayes RB, Winn DM, Bravo-Otero E, Blot WJ, Kleinman DV, Brown LM, Armenian HK, Fraumeni JJ, Shields PG (1997). Alcohol dehydrogenase 3 genotype and risk of oral cavity and pharyngeal cancers. J Natl Cancer Inst.

[116] Olshan AF, Weissler MC, Watson MA, Bell DA (2000). GSTM1, GSTT1, GSTP1, CYP1A1, and NAT1 polymorphisms, tobacco use, and the risk of head and neck cancer. Cancer Epidemiol Biomarkers Prev.

[117] BURNET M (1957). Cancer: a biological approach. III. Viruses associated with neoplastic conditions. IV. Practical applications. Br Med J.

[118] Corthay A (2014). Does the immune system naturally protect against cancer?. Front Immunol.

[119] Shankaran V, Ikeda H, Bruce AT, White JM, Swanson PE, Old LJ, Schreiber RD (2001). IFNgamma and lymphocytes prevent primary tumour development and shape tumour immunogenicity. Nature.

[120] Swann JB, Smyth MJ (2007). Immune surveillance of tumors. J Clin Invest.

[121] Dubrow R, Silverberg MJ, Park LS, Crothers K, Justice AC (2012). HIV infection, aging, and immune function: implications for cancer risk and prevention. Curr Opin Oncol.

[122] Hadden JW (2003). Immunodeficiency and cancer: prospects for correction. Int Immunopharmaco.

[123] Shiels MS, Cole SR, Kirk GD, Poole C (2009). A meta-analysis of the incidence of non-AIDS cancers in HIV-infected individuals. J Acquir Immune Defic Syndr.

[124] Beachler DC, D'souza G (2013). Oral human papillomavirus infection and head and neck cancers in HIV-infected individuals. Curr Opin Oncol.

[125] Kreimer AR, Alberg AJ, Daniel R, Gravitt PE, Viscidi R, Garrett ES, Shah KV, Gillison ML (2004). Oral human papillomavirus infection in adults is associated with sexual behavior and HIV serostatus. J Infect Dis.

[126] Beachler DC, Weber KM, Margolick JB, Strickler HD, Cranston RD, Burk RD, Wiley DJ, Minkoff H, Reddy S, Stammer EE, Gillison ML, D'souza G (2012). Risk factors for oral HPV infection among a high prevalence population of HIV-positive and at-risk HIV-negative adults. Cancer Epidemiol Biomarkers Prev.

[127] Cameron JE, Mercante D, O'Brien M, Gaffga AM, Leigh JE, Fidel PJ, Hagensee ME (2005). The impact of highly active antiretroviral therapy and immunodeficiency on human papillomavirus infection of the oral cavity of human immunodeficiency virus-seropositive adults. Sex Transm Dis.

[128] Rabinovics N, Mizrachi A, Hadar T, Ad-El D, Feinmesser R, Guttman D, Shpitzer T, Bachar G (2014). Cancer of the head and neck region in solid organ transplant recipients. Head Neck.

[129] Engels EA, Pfeiffer RM, Fraumeni JJ, Kasiske BL, Israni AK, Snyder JJ, Wolfe RA, Goodrich NP, Bayakly AR, Clarke CA, Copeland G, Finch JL, Fleissner ML, Goodman MT, Kahn A, Koch L (2011). Spectrum of cancer risk among US solid organ transplant recipients. JAMA.

[130] Duvoux C, Delacroix I, Richardet JP, Roudot-Thoraval F, Metreau JM, Fagniez PL, Dhumeaux D, Cherqui D (1999). Increased incidence of oropharyngeal squamous cell carcinomas after liver transplantation for alcoholic cirrhosis. Transplantation.

[131] Deeb R, Sharma S, Mahan M, Al-Khudari S, Hall F, Yoshida A, Schweitzer V (2012). Head and neck cancer in transplant recipients. Laryngoscope.

[132] Chak E, Saab S (2010). Risk factors and incidence of de novo malignancy in liver transplant recipients: a systematic review. Liver Int.

[133] Scheifele C, Reichart PA, Hippler-Benscheidt M, Neuhaus P, Neuhaus R (2005). Incidence of oral, pharyngeal, and laryngeal squamous cell carcinomas among 1515 patients after liver transplantation. Oral Oncol.

[134] Nelissen C, Lambrecht M, Nevens F, Van Raemdonck D, Vanhaecke J, Kuypers D, Pirenne J, Nuyts S (2014). Noncutaneous head and neck cancer in solid organ transplant patients: single center experience. Oral Oncol.

[135] Ohman J, Rexius H, Mjornstedt L, Gonzalez H, Holmberg E, Dellgren G, Hasseus B (2015). Oral and lip cancer in solid organ transplant patients—a cohort study from a Swedish Transplant Centre. Oral Oncol.

[136] Gourin CG, Terris DJ (2004). Head and neck cancer in transplant recipients. Curr Opin Otolaryngol Head Neck Surg.

[137] Pollard JD, Hanasono MM, Mikulec AA, Le QT, Terris DJ (2000). Head and neck cancer in cardiothoracic transplant recipients. Laryngoscope.

[138] Coordes A, Albers AE, Lenarz M, Seehofer D, Puhl G, Pascher A, Neuhaus R, Neuhaus P, Pratschke J, Andreou A (2016). Incidence and long-term survival of patients with de novo head and neck carcinoma after liver transplantation. Head Neck.

[139] Santos-Silva AR, Carvalho AM, Jorge J, Almeida OP, Vargas PA, Lopes MA (2014). Tongue squamous cell carcinoma in young nonsmoking and nondrinking patients: 3 clinical cases of orthodontic interest. Am J Orthod Dentofacial Orthop.

[140] MOORE C (1958). Visceral squamous cancer in children. Pediatrics.

[141] Amichetti M (1989). Squamous cell carcinoma of the oral tongue in patients less than fifteen years of age. Report of a case and review of the literature. J Craniomaxillofac Surg.

[142] Newman AN, Rice DH, Ossoff RH, Sisson GA (1983). Carcinoma of the tongue in persons younger than 30 years of age. Arch Otolaryngol.

[143] Park EJ, Lee JH, Yu GY, He G, Ali SR, Holzer RG, Osterreicher CH, Takahashi H, Karin M (2010). Dietary and genetic obesity promote liver inflammation and tumorigenesis by enhancing IL-6 and TNF expression. Cell.

[144] Patel DR (1999). Smoking and children. Indian J Pediatr.

[145] Bill TJ, Reddy VR, Ries KL, Gampper TJ, Hoard MA (2001). Adolescent gingival squamous cell carcinoma: Report of a case and review of the literature. Oral Surg Oral Med Oral Pathol Oral Radiol Endod.

[146] Wechsler H, Nelson TF (2001). Binge drinking and the American college student: what's five drinks?. Psychol Addict Behav.

[147] Toner M, O'Regan EM (2009). Head and neck squamous cell carcinoma in the young: a spectrum or a distinct group? Part 1. Head Neck Pathol.

[148] Bui TC, Markham CM, Ross MW, Mullen PD (2013). Examining the association between oral health and oral HPV infection. Cancer Prev Res (Phila).

[149] Ahrens W, Pohlabeln H, Foraita R, Nelis M, Lagiou P, Lagiou A, Bouchardy C, Slamova A, Schejbalova M, Merletti F, Richiardi L, Kjaerheim K, Agudo A, Castellsague X, Macfarlane TV, Macfarlane GJ (2014). Oral health, dental care and mouthwash associated with upper aerodigestive tract cancer risk in Europe: the ARCAGE study. Oral Oncol.

[150] Skrivele S, Care R, Berzina S, Kneist S, de Moura-Sieber V, de Moura R, Borutta A, Maslak E, Tserekhava T, Shakovets N, Wagner M (2013). Caries and its risk factors in young children in five different countries. Stomatologija.

